# The relationship between longevity and diet is genotype dependent and sensitive to desiccation in *Drosophila melanogaster*

**DOI:** 10.1242/jeb.230185

**Published:** 2020-12-02

**Authors:** Andrew W. McCracken, Eleanor Buckle, Mirre J. P. Simons

**Affiliations:** Department of Animal and Plant Sciences & Bateson Centre, The University of Sheffield, Sheffield S10 2TN, UK

**Keywords:** Ageing, Dietary restriction, Fruit fly, Overfeeding, Reaction norm, Starvation

## Abstract

Dietary restriction (DR) is a key focus in ageing research. Specific conditions and genotypes were recently found to negate lifespan extension by DR, questioning its universal relevance. However, the concept of dietary reaction norms explains why the effects of DR might be obscured in some situations. We tested the importance of dietary reaction norms by measuring longevity and fecundity on five diets in five genotypes, with and without water supplementation in female *Drosophila melanogaster* (*N*>25,000). We found substantial genetic variation in the response of lifespan to diet. Flies supplemented with water rescued putative desiccation stress on the richest diets, suggesting that water availability can be an experimental confound. Fecundity declined on these richest diets, but was unaffected by water, and this reduction is thus most likely to be caused by nutritional toxicity. Our results demonstrate empirically that a range of diets need to be considered to conclude an absence of the DR longevity effect.

## Abstract (precis)

**Summary:** Lifespan extension under dietary restriction can occasionally be obscured. In *Drosophila melanogaster*, a robust appreciation of dietary reaction norms is necessary to conclude an absence of the dietary restriction longevity effect.

## INTRODUCTION

**Fig. 1.**
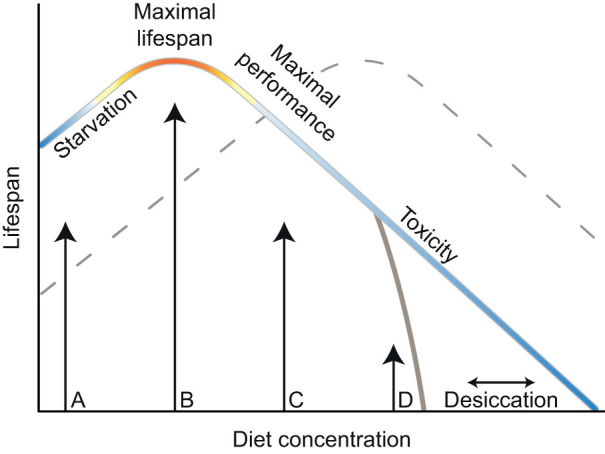
**Schematic of multiple thresholds in the lifespan reaction norm to diet.** Diet concentration has a bell-shaped relationship with lifespan, ranging from malnutrition (A), dietary restriction (B) and maximal performance – or highest Darwinian fitness – at a relatively rich diet (C) to overfeeding, leading to nutritional toxicity (D). As a detailed reaction norm is rarely known, a dietary dyad (although often used) can lead to misleading conclusions. A dietary dyad (A and C) can show no response at all owing to the symmetry in the shape of the reaction norm. Furthermore, genetic or environmental effects can alter the shape or shift the reaction norm (dashed line), or lead to effects at only specific parts of the reaction norm (continuous grey line, e.g. desiccation). For example, diets B and C result in a dietary restriction response on the focal curve, but malnutrition on the dashed curve.

Dietary restriction (DR), the limitation of food intake but avoidance of malnutrition, extends an organism's lifespan. The generality of the DR response has been questioned, however, by reports that DR does not extend lifespan under certain experimental conditions (Austad, 2012; Dick et al., 2011; Ja et al., 2009; Piper et al., 2010) or in a considerable proportion of the genotypes tested (Dick et al., 2011; Jin et al., 2020; Liao et al., 2010; Mitchell et al., 2016; Rikke et al., 2010; Swindell, 2012; Wilson et al., 2020). These conclusions are routinely based upon experiments using two diets (dietary dyad) alone, whereas it is recognised that a change in the continuous relationship between diet and lifespan (reaction norm) can obscure lifespan extension by DR (Flatt, 2014; Tatar, 2011). The bell-shaped nature of the dietary reaction norm dictates that one particular diet concentration, in one genotype or environment, will result in the longest lifespan; lower or higher diet concentrations will induce a shortened lifespan due to malnutrition or overfeeding, respectively. Where a particular dietary dyad falls on this reaction norm will determine the magnitude of the DR effect and can even lead to the erroneous conclusion that DR shortens lifespan (Fig. 1).

Few studies have examined dietary reaction norms in more detail by titrating the supply of protein or calories across multiple genotypes or environments, and none has tested both genetic and environmental effects on dietary reaction norms simultaneously. Of these studies, a fraction employed transgenic or laboratory strains (Clancy et al., 2002; Grandison et al., 2009; Min et al., 2008; Skorupa et al., 2008; Tatar, 2011; Wang et al., 2009) and demonstrated varying degrees of genetic variance in the plastic response to diet. Across these studies, shifts in dietary reaction norms on the *x*- or *y*-plane are more apparent than changes in the overall shape of the relationship between diet and longevity (Flatt, 2014; Tatar, 2011). Whether genetic variation in transgenic and laboratory strain experiments is representative of standing genetic variation of natural populations is, however, unclear. A naturalistic appreciation of the genetic variation of the DR response becomes particularly important when null responses are interpreted to question the universal properties of DR important in translating its benefits to our own species. One previous study did measure detailed reaction norms using wild-derived outbred populations and found a degree of genetic variance for the relationship between diet and lifespan (Metaxakis and Partridge, 2013). However, the estimate of genetic variance of a population level trait, such as lifespan, when estimated from between outbred stains (Whitlock and Fowler, 1999) will be affected by mortality heterogeneity (Chen et al., 2013), which can bias the estimated level of genetic variance upwards or downwards.

When specific environmental effects interact or interfere with the DR reaction norm, the use of dietary dyads – or the neglect of environmental confounds, like desiccation – could similarly lead to misleading conclusions. For flies specifically, water supplementation has been suggested to diminish the effect of DR on lifespan (Dick et al., 2011; Ja et al., 2009). The conclusion that water completely explains DR has been discredited (Piper et al., 2010), but flies nonetheless value water as a resource and consume 1–2 μl per day, with higher consumption at higher dietary yeast (Fanson et al., 2012) and sugar concentrations (van Dam et al., 2020). Hence, erroneous conclusions could be drawn from diet responses if desiccation presents a genotype- or diet-specific hazard.

Here, we present DR reaction norms for fecundity and longevity across five genotypes in female flies (*Drosophila melanogaster*) with and without water supplementation using high sample sizes. We show empirically across five wild-derived, inbred lines that there are strong genetic and environmental elements to dietary reaction norms, and therefore the thorough appreciation of reaction norms is critical when interpreting diet effects across genotypes and environments.

## MATERIALS AND METHODS

### Fly husbandry, experimental protocol and dietary regimes

For lifespan experiments, adult *Drosophila* *melanogaster* Meigen 1830 were provided with either 0.5, 2, 5, 8 or 14% autolysed yeast medium. All other medium components [13% table sugar, 6% cornmeal, 1% agar and 0.225% (w/v) nipagin] remained the same, given that the dietary protein axis is the main lifespan determinant in flies (Jensen et al., 2015; Lee et al., 2008). Note, cornmeal concentration was halved in 14% yeast medium to allow dispensing of this medium. Halving cornmeal concentration in all diets would have affected viscosity of the medium at lower yeast concentrations, possibly resulting in yeast granules settling at the bottom of vials, and would have made our diets less comparable to our own previous work (McCracken et al., 2020). Full cornmeal concentration 14% diets, we speculate, would have intensified, rather than have relieved, desiccative stress at this yeast concentration. Statistical analyses and figures consider our diets to be nominal (categorical) measurements, and do not imply a fixed degree of difference attributable to yeast concentration. Purpose-built demography cages included two openings, one for the supplementation of food, and one for water–agar (2% agar) or empty vial. Cages contained between 70 and 125 females each (mode of ∼100 females), with five cages per treatment, per genotype (*N*=50 cages per genotype). For one genotype, DGRP-195, sample size was even higher: an additional two cages of water-supplemented and control cages with 2% medium. All experimental flies were reared and mated on 8% medium for 48 h, and kept in cages on 8% medium until age 3–4 days, when experimental dietary treatments started. Flies were scored every 48 h, where dead flies were removed and counted, and food vials were replaced.

To establish dietary reaction responses, flies were exposed to continuous diets with the addition, or absence, of water–agar supplementation. To test the effect of water supplementation on longevity, we provided an additional vial of water–agar (‘water supplementation’), or an empty vial (‘control’), to each cage. Separation of food and water sources allowed flies to choose their source of nourishment, and eliminated the need for hydration to be coupled with caloric intake. Dietary treatments were balanced for age and date of eclosion. All flies presented were grown within one batch. The experiment was carried out on a small collection of DGRP lines (Mackay et al., 2012; DGRP-195, -217, -239, -362 and -853), which were generated through full-sibling mating of wild-type females in 2003. These lines were a subset of the lines we used in McCracken et al. (2020), where we observed different responses comparing 2 and 8% yeast diets. Previously observed responses of the five lines to 2% yeast diets – either typical, or starvation – were replicated in the results presented here.

### Fecundity

Feeding vials were imaged and analysed using image analysis software QuantiFly (Waithe et al., 2015) to determine the relative quantity of egg laying. Egg counts based on image recognition do not necessarily provide an absolute count, as with manual egg counting, but are suitable for comparative estimates. The combined estimate achieved using image analysis has the advantage of using egg laying from many females in the same vial, averaging out biological variation between females. Vials were removed, during normal scoring periods, from all cages containing eggs from flies aged 11 or 12 days.

### Data analysis

For survival analysis, mixed Cox proportional hazard models were used that included ‘cage’ as a random term to correct for uncertainty of pseudoreplicated effects within demography cages (Ripatti and Palmgren, 2000; Therneau et al., 2003). Additional specific tests of coefficients are provided that combine the single and interaction term (in a *Z*-test, using the maximum s.e.m. of the factors compared) to test how survival was changing in water-treated flies, compared with respective control treatments. Note that formal tests for proportionality of hazards are not available for mixed-effects Cox regressions. For survival data comparisons, we report the full model, and models fitted within each genotype separately (see Tables S1–S12). By splitting the analysis between genotypes, bias introduced by deviations in proportionality of hazards between genotypes is avoided. Qualitative conclusions remain similar, irrespective of how these models are fitted. Interpretations from the Cox mixed-effects model are based on a full model including the three-way interaction between diet, water supplementation and genotype. Coefficients are reported as logged hazards with significance based on *Z*-tests. Right-censoring was included, and dietary treatments were considered categorical factors.

Egg laying was analysed using linear models of log-transformed fecundity count data. Flies only differed by 1 day in age, and age was equally distributed across treatments and measured in a balanced design. Bayesian information criterion with backward elimination of terms was used for model comparisons and selection, and resulted in a model that contained the terms, and interaction between genotype and diet. Water was added to our models to directly test for any effect on fecundity, but this proved negligible (Tables S13 and S14).

For hazard ratio figures, ratios are plotted as coefficients derived from within-line Cox mixed-effects models, with error bars representing 95% confidence intervals.

## RESULTS AND DISCUSSION

**Fig. 2.**
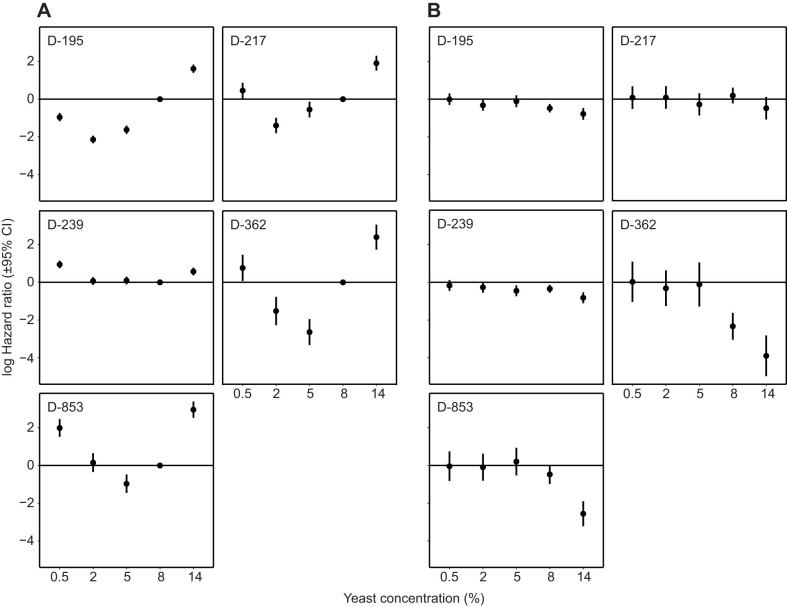
**Log hazard ratios of diet and water supplementation in a panel of** **DGRP genotypes.** (A) Dietary reaction norms vary in a genotype-specific manner. (B) Water supplementation, relative to control treatment, rescues desiccation in a diet- and genotype-dependent manner. Hazard ratios represent risk of dying; therefore, higher values indicate shorter lifespans and are relative. Ratios are plotted as coefficients derived from within-line Cox mixed-effects models, with error bars representing 95% confidence intervals (CI). For A, 8% yeast treatment was treated as a reference, and as such, no CIs are available. Rates here are relative to 8% yeast diet, and lines represent this standard; *N*=25,519 females in total, and 4800–5282 per genotype. For B, hazard rates are relative to the corresponding control for each diet. Horizontal lines represent a water effect size of 0; *N*=12,737 females in total, and 2396–2629 per genotype.

The genotypes tested showed a classical bell-shaped response to diet. Longer lifespans were observed at intermediate dietary yeast concentrations, consistent with DR (*P*<0.001; Fig. 2A, Fig. S4, Tables S1–S6). All genotypes also exhibited a reduction in survival at very lowest yeast concentrations (starvation), and at the very highest concentration (maximal performance or nutritional toxicity). We detected considerable genetic variation in the response to diet (genotype×diet; χ^2^=162, d.f.=16, *P*<0.001) with the diet of maximum longevity and the magnitude of the diet response differing between genotypes (Fig. 2). This result held even upon the exclusion of our highest yeast concentration diet (χ^2^=217, d.f.=12, *P*<0.001).

To test the effect of desiccation, we compared longevity under control conditions with water-supplemented conditions. Supplemental water reduced mortality particularly at higher yeast concentrations, and we found genetic variance for this environmental effect (genotype×diet×water; χ^2^=160, d.f.=16, *P*<0.001; Fig. 2B, Tables S2–S6). At the highest yeast concentrations, this amounted to a 1.5- to 50-fold reduction in hazard rate. This result also held when excluding our highest yeast concentration diet (χ^2^=75, d.f.=12, *P*<0.001). Given this, particular caution should be taken when considering the effect of desiccation, especially in organisms without *ad libitum* access to water and when fed a concentrated diet. To assess statistically whether water supplementation abolished DR-induced life extension (Ja et al., 2009; Piper et al., 2010) we ran our statistical models within the water treatment only, but found no evidence for this suggestion (Fig. S1; Tables S7–S12). The observed mortality can thus be partitioned into nutrition- or hydration-based causes. We therefore conclude that desiccation can play an experimentally confounding role in DR, but is not causal in the link between nutrition and longevity, as the removal of desiccation as a variable does not eliminate the longevity response to diet.

DR is known to reduce reproductive output and is commonly interpreted as a response to decreased energy availability (Moatt et al., 2016). The effect of overfeeding on reproduction, although appreciated in humans (Broughton and Moley, 2017), has received little attention (McCracken et al., 2020). These two responses were evident in egg laying: an increase with yeast concentration, and a stabilisation or decline at the highest yeast concentrations (Figs S2 and S3, Tables S13 and S14). As with mortality, genetic lines also differed in the reproductive response to diet (*F*=6.3, d.f.=16, *P*<0.001). Reduced egg laying together with a reduction in survival lowered predicted lifetime reproductive fitness at the richest diet (Fig. S3). Egg laying was not affected by water supplementation (Fig. S2, Tables S13 and S14; *P*>0.15). Notably, even when water rescued mortality caused by desiccation at the high yeast concentrations, egg laying was unaffected (Fig. 2, Fig. S2). Given this, we infer that the decline in reproductive output at the highest yeast concentration was not due to desiccation stress, but to nutritional toxicity. By contrast, the rescue of mortality at high yeast concentrations by water supplementation is therefore likely to be separate and driven by desiccation. However, as the reduction in fecundity was only observed in our highest yeast concentration diet, and this was the only diet in which cornmeal concentration was halved (see Materials and Methods), it is possible that the reduction in cornmeal acts as a nutritional limiter of reproductive output, and this will require further testing.

In conclusion, we observed significant genetic and environmentally induced variation in the lifespan and fecundity responses to diet. Our data used females only, but similar effects in males could explain observations of sexual dimorphism in the response to diet and likewise requires investigation (Camus et al., 2017; Jensen et al., 2015; Regan et al., 2016). These data now directly demonstrate that specific care is needed when interpreting effects of DR across genotypes, experimental conditions or environments. We acknowledge that carrying out full reaction norms in all DR experiments would be laborious, especially in mammalian models (reviewed in Selman and Swindell, 2018). However, it is increasingly acknowledged that personalising the degree of DR to genotype or environment will be key to translating the benefits of DR to humans (Perez-Matos and Mair, 2020). When genetic variance in DR is the object of study, we suggest selecting dietary dyads that differ only minimally when genetic variance in DR is the object of study. Such a strategy reduces the chance that tested diets diverge considerably from maximal lifespans, leading to starvation or nutritional toxicity (Fig. 1). Furthermore, we suggest when environmental conditions, such as water (Ja et al., 2009), sex (Regan et al., 2016) and microbiome (Wong et al., 2014) are presumed to negate the DR response, that a *post hoc* reaction norm is performed. Similar considerations hold for mechanistic research. Should, for example, a genetic manipulation remove the DR response, only a full dietary reaction norm can demonstrate how such an effect arises: by either a shift in, or compression of, the reaction norm (Flatt, 2014; Tatar, 2011). The importance of reaction norms when studying DR has been stressed before, but this is the first high sample size data across multiple wild-type inbred genotypes and diets, including an environmental confound, that demonstrates this empirically.

## Supplementary Material

Supplementary information
