# Supplementary material for: The relationship between longevity and diet is genotype dependent and sensitive to desiccation in Drosophila melanogaster
Source: J Exp Biol. 2020 Dec 2;223(23):jeb230185. doi: 10.1242/jeb.230185 (PMC7725603; doi:10.1242/jeb.230185)
Supplement: Supplementary information [file jexbio-223-230185-s1.pdf]

Supplementary information

Figures

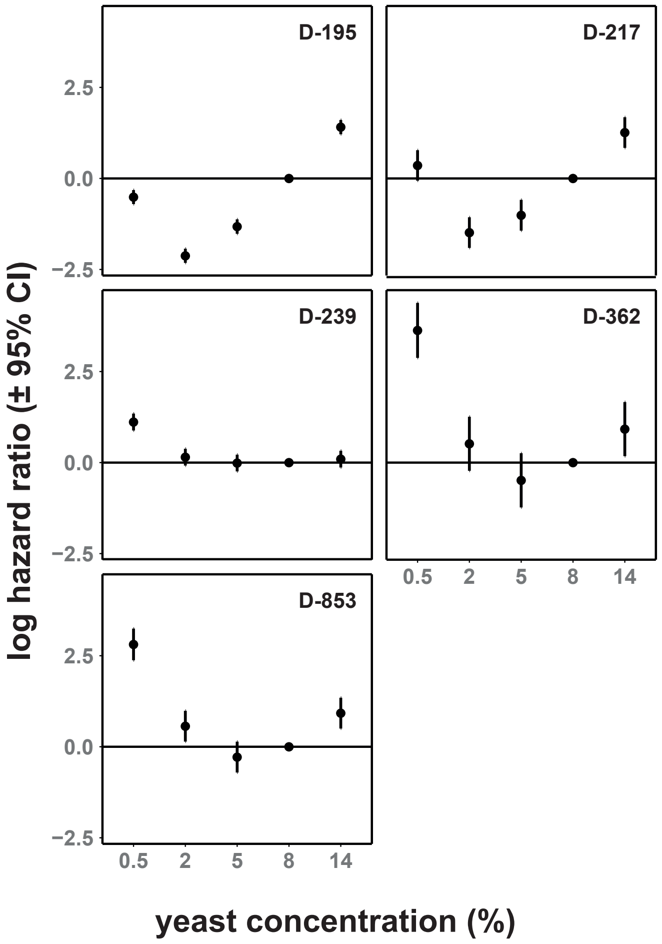

Fig. S1. Log hazard ratios of diet within water-treated cages in a panel of DGRP genotypes. Reaction norms to diet still differ in water-treated circumstances. Hazard ratios represent the inverse of typical survival reaction norms to diet. 8% yeast treatment was treated as a reference and as such, no CIs are available. Rates here are relative to 8% yeast diet, and lines represent this standard. N = 12,737 females total; 2,396-2629 per genotype. Hazard ratios have the benefit over median lifespan in that they are directly related to the appropriate statistics used for time-to-event data. In addition, they are directly comparable in a quantitative fashion across genotypes of different lifespans, as they express a relative risk.

Journal of Cell Science • Supplementary information

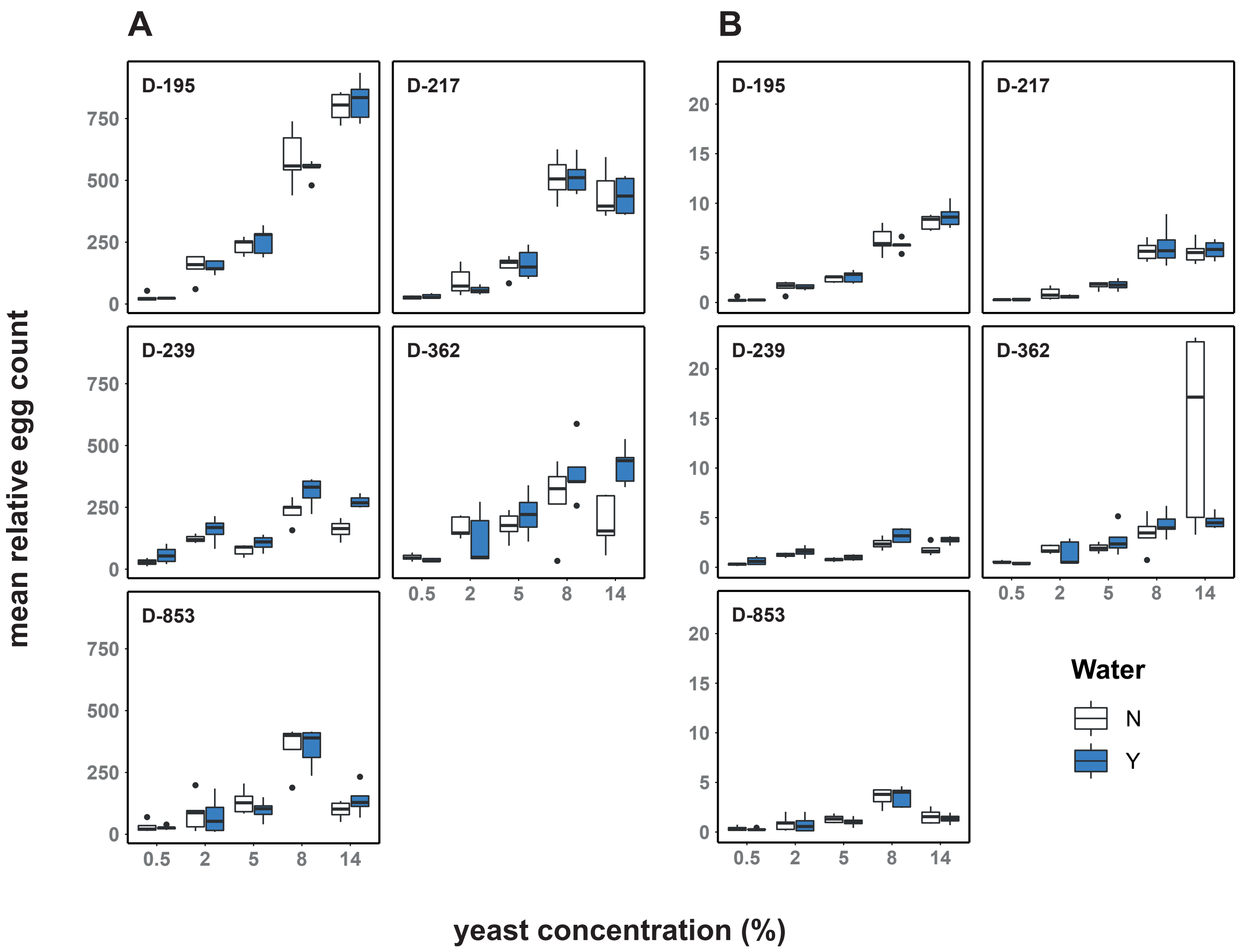

Fig. S2. Fecundity analysis of panel under all conditions. Fecundity has a positive relationship with dietary yeast concentration, except at the highest yeast concentration assayed (14%) for most genotypes. **A** - raw egg counts. **B** - mortality-corrected counts. Counts generated using QuantiFly software. Counts are relative, but directly comparable. Flies assayed at age 11-12 days, with boxplots aggregating totals (median, with the box depicting a quartile each way, and whiskers showing the range; outliers plotted as dots). Each cage was assayed on 1 scoring day at this age. Mortality corrected counts (**B**) generated by dividing raw counts, by N flies remaining in cage at the time of assaying. N = 25,519 females total; 4,800-5,282 per genotype. Note that DGRP-362 experienced significant mortality at this age under 14% yeast dietary treatment. This is the cause of the discrepancy in significance between raw, and age-adjusted fecundity counts. Note, egg-laying was not assessed throughout life and in natural circumstances lifespan of the fly is truncated by extrinsic factors, e.g. predation. We nonetheless, tentatively conclude that the enhanced mortality and reduced egg laying on very rich diets is caused by nutritional toxicity.

Journal of Cell Science • Supplementary information

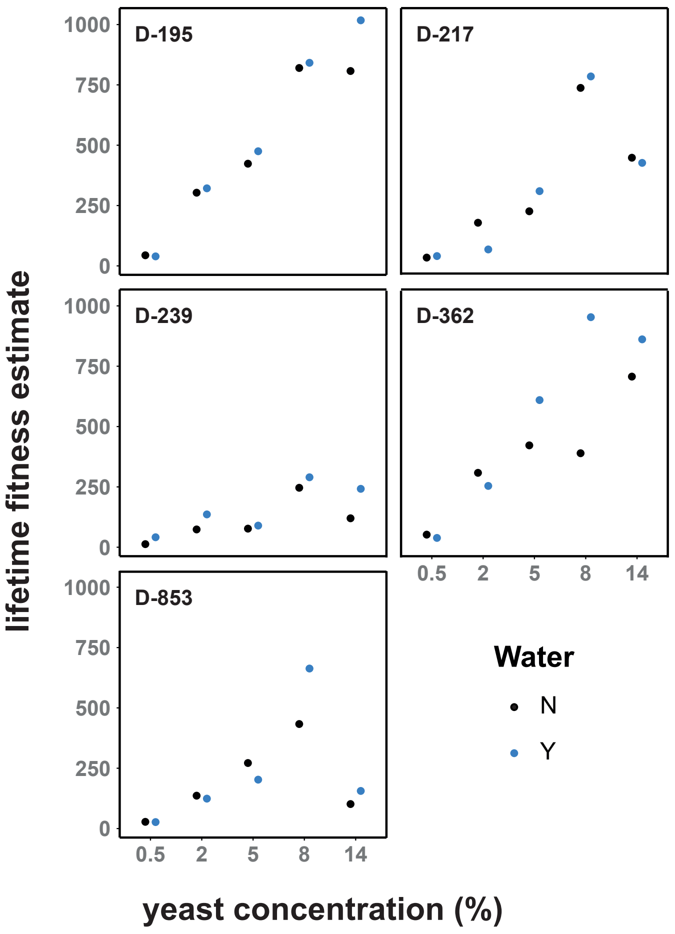

Fig. S3. Lifetime reproductive fitness estimates of panel under all conditions. Lifetime fitness has a positive relationship with dietary yeast concentration, except at the highest yeast concentration assayed (14%) for most genotypes. Mortality-adjusted egg counts from Fig. S2 were multiplied by the area under the relevant survival curve (restricted mean) to generate lifetime estimates. N = 25,519 females total; 4,800-5,282 per genotype.

Journal of Cell Science • Supplementary information

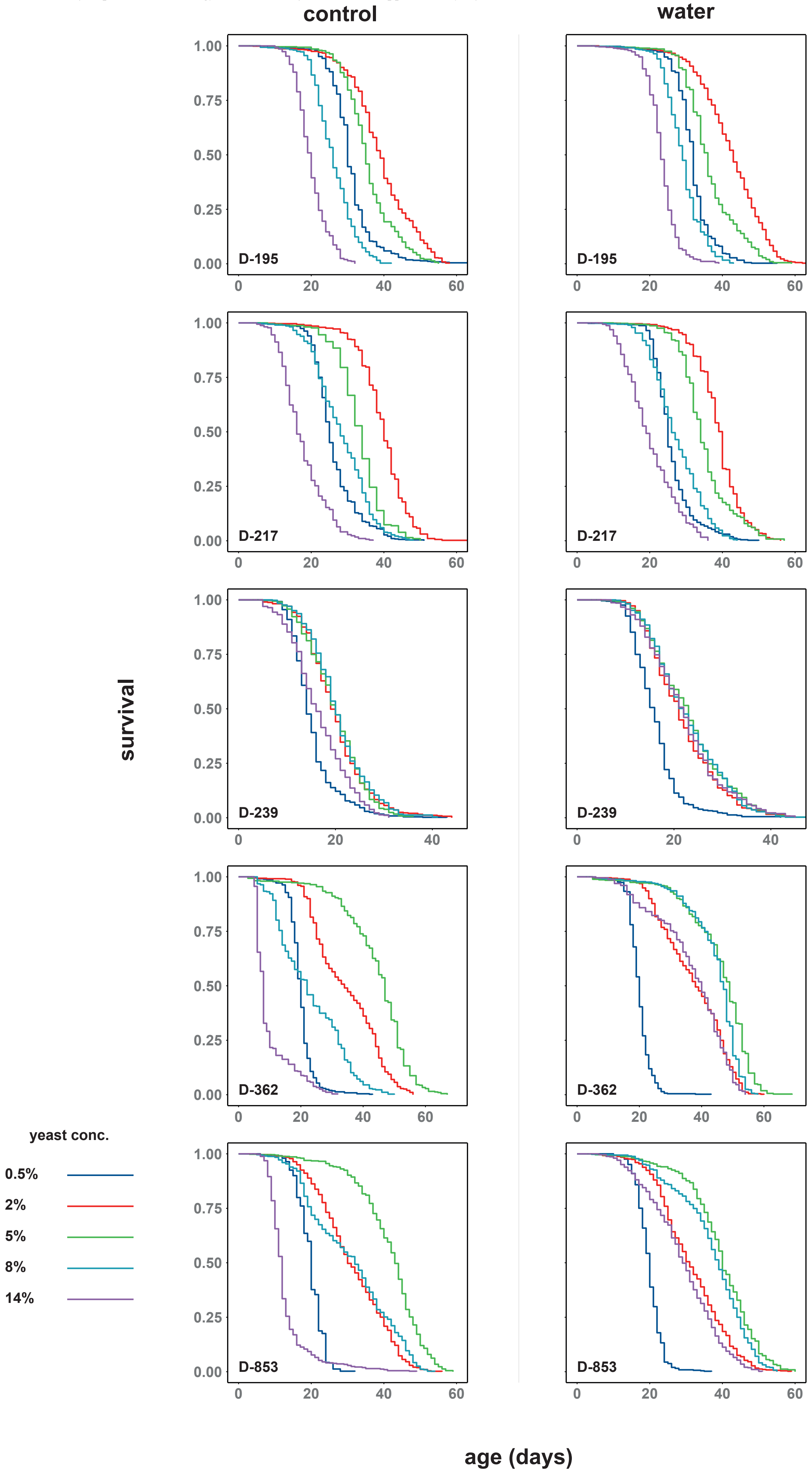

Journal of Cell Science • Supplementary information

Fig. S4. Survival curves of panel in response to diet. Dietary reaction norms vary in a genotype-specific manner. Survival curves are separated by genotype, and water-supplementation status. N = 25,519 females total; 4,800-5,282 per genotype.

Journal of Cell Science • Supplementary information

Tables

Table S1. Effect of diet and water supplementation on mortality across 5 DGRP lines (DGRP-195 is reference).

	Full Model			
coefficient	estimate	exp	s.e.	p
water	-0.390	0.677	0.228	0.086
0.5% yeast	-0.796	0.451	0.228	<0.001
2% yeast	-1.856	0.156	0.206	<0.001
5% yeast	-1.296	0.274	0.225	<0.001
14% yeast	1.043	2.837	0.226	<0.001
217	-0.444	0.641	0.226	0.05
239	0.763	2.145	0.227	0.001
362	0.104	1.110	0.227	0.645
853	-1.090	0.336	0.223	<0.001
0.5% yeast * water	0.470	1.601	0.322	0.144
2% yeast * water	0.030	1.031	0.300	0.92
5% yeast * water	0.277	1.319	0.322	0.39
14% yeast * water	-0.156	0.856	0.322	0.629
217 * 0.5% yeast	1.195	3.302	0.320	<0.001
217 * 2% yeast	0.632	1.881	0.310	0.042
217 * 5% yeast	0.786	2.195	0.321	0.014
217 * 14% yeast	0.643	1.903	0.322	0.046
239 * 0.5% yeast	1.793	6.008	0.321	<0.001
239 * 2% yeast	1.887	6.599	0.308	<0.001
239 * 5% yeast	1.429	4.174	0.321	<0.001
239 * 14% yeast	-0.382	0.683	0.324	0.238
362 * 0.5% yeast	1.754	5.775	0.319	<0.001
362 * 2% yeast	0.262	1.300	0.311	0.399
362 * 5% yeast	-1.322	0.267	0.315	<0.001
362 * 14% yeast	1.880	6.551	0.316	<0.001
853 * 0.5% yeast	3.087	21.901	0.318	<0.001
853 * 2% yeast	2.029	7.604	0.307	<0.001
853 * 5% yeast	0.287	1.332	0.320	0.37
853 * 14% yeast	2.100	8.168	0.320	<0.001
217 * water	0.551	1.736	0.322	0.087
239 * water	-0.101	0.904	0.322	0.754
362 * water	-2.015	0.133	0.319	<0.001
853 * water	-0.101	0.904	0.323	0.753
217 * 0.5% yeast * water	-0.568	0.567	0.455	0.212
217 * 2% yeast * water	-0.114	0.893	0.441	0.797
217 * 5% yeast * water	-0.702	0.496	0.456	0.123
217 * 14% yeast * water	-0.448	0.639	0.456	0.327
239 * 0.5% yeast * water	-0.145	0.865	0.457	0.751
239 * 2% yeast * water	0.126	1.134	0.440	0.775
239 * 5% yeast * water	-0.427	0.652	0.455	0.348
239 * 14% yeast * water	-0.383	0.682	0.457	0.402
362 * 0.5% yeast * water	1.990	7.319	0.456	<0.001
362 * 2% yeast * water	2.068	7.912	0.439	<0.001
362 * 5% yeast * water	2.007	7.444	0.457	<0.001
362 * 14% yeast * water	-1.942	0.143	0.451	<0.001
853 * 0.5% yeast * water	-0.027	0.974	0.458	0.954
853 * 2% yeast * water	0.351	1.420	0.441	0.427
853 * 5% yeast * water	0.433	1.541	0.457	0.344
853 * 14% yeast * water	-2.056	0.128	0.455	<0.001

Journal of Cell Science • Supplementary information

Table S2. Effect of diet and water supplementation on mortality within DGRP-195.

	Estimates from individual model				Effect of water, compared to no water		
coefficient	estimate	exp	s.e.	p	estimate	exp	p
water supplementation	-0.481	0.618	0.113	<0.001			
0.5% yeast	-0.954	0.385	0.112	<0.001			
2% yeast	-2.139	0.118	0.103	<0.001			
5% yeast	-1.620	0.198	0.112	<0.001			
14% yeast	1.612	5.012	0.112	<0.001			
0.5% yeast * water	0.475	1.608	0.158	0.003	-0.006	0.994	0.971
2% yeast * water	0.156	1.169	0.147	0.289	-0.324	0.723	0.028
5% yeast * water	0.369	1.446	0.160	0.021	-0.112	0.894	0.483
14% yeast * water	-0.301	0.740	0.159	0.059	-0.781	0.458	<0.001

Table S3. Effect of diet and water supplementation on mortality within DGRP-217.

	Estimates from individual model				Effect of water, compared to no water		
coefficient	estimate	exp	s.e.	p	estimate	exp	p
water supplementation	0.190	1.209	0.213	0.373			
0.5% yeast	0.454	1.574	0.211	0.032			
2% yeast	-1.395	0.248	0.208	<0.001			
5% yeast	-0.549	0.578	0.212	0.01			
14% yeast	1.905	6.716	0.200	<0.001			
0.5% yeast * water	-0.108	0.898	0.306	0.724	0.082	1.08	0.789
2% yeast * water	-0.105	0.901	0.306	0.732	0.085	1.08	0.781
5% yeast * water	-0.470	0.625	0.300	0.117	-0.280	0.75	0.350
14% yeast * water	-0.668	0.513	0.306	0.029	-0.479	0.62	0.118

Table S4. Effect of diet and water supplementation on mortality within DGRP-239.

	Estimates from individual model				Effect of water, compared to no water		
coefficient	estimate	exp	s.e.	p	estimate	exp	p
water supplementation	-0.343	0.710	0.105	0.001			
0.5% yeast	0.946	2.575	0.104	<0.001			
2% yeast	0.070	1.072	0.104	0.502			
5% yeast	0.094	1.098	0.103	0.363			
14% yeast	0.572	1.771	0.103	<0.001			
0.5% yeast * water	0.173	1.188	0.148	0.243	-0.170	0.843	0.249
2% yeast * water	0.079	1.082	0.148	0.593	-0.264	0.768	0.075
5% yeast * water	-0.108	0.898	0.147	0.464	-0.451	0.637	0.002
14% yeast * water	-0.476	0.621	0.147	0.001	-0.819	0.441	<0.001

Table S5. Effect of diet and water supplementation on mortality within DGRP-362.

	Estimates from individual model				Effect of water, compared to no water		
coefficient	estimate	exp	s.e.	p	estimate	exp	p
water supplementation	-2.330	0.097	0.363	<0.001			
0.5% yeast	0.763	2.144	0.356	0.032			
2% yeast	-1.520	0.219	0.383	<0.001			
5% yeast	-2.638	0.072	0.351	<0.001			
14% yeast	2.393	10.950	0.339	<0.001			
0.5% yeast * water	2.358	10.571	0.545	<0.001	0.028	1.029	0.958
2% yeast * water	2.016	7.509	0.482	<0.001	-0.314	0.731	0.515
5% yeast * water	2.213	9.145	0.597	<0.001	-0.116	0.890	0.845
14% yeast * water	-1.564	0.209	0.549	0.004	-3.894	0.020	<0.001

Journal of Cell Science • Supplementary information

Table S6. Effect of diet and water supplementation on mortality within DGRP-853.

	Estimates from individual model				Effect of water, compared to no water		
coefficient	estimate	exp	s.e.	p	estimate	exp	p
water supplementation	-0.473	0.623	0.259	0.067			
0.5% yeast	1.987	7.293	0.239	<0.001			
2% yeast	0.150	1.162	0.254	0.554			
5% yeast	-0.967	0.380	0.248	<0.001			
14% yeast	2.955	19.204	0.223	<0.001			
0.5% yeast * water	0.436	1.547	0.399	0.274	-0.037	0.964	0.926
2% yeast * water	0.379	1.461	0.365	0.299	-0.094	0.910	0.797
5% yeast * water	0.680	1.973	0.373	0.068	0.206	1.229	0.58
14% yeast * water	-2.083	0.125	0.339	<0.001	-2.556	0.078	<0.001

Table S7. Effect of diet on mortality across 5 water-supplemented DGRP lines (DGRP-195 is reference).

	Full Model			
coefficient	estimate	exp	s.e.	p
0.5% yeast	-0.345	0.709	0.228	0.131
2% yeast	-1.921	0.146	0.202	<0.001
5% yeast	-1.060	0.347	0.224	<0.001
14% yeast	0.930	2.535	0.225	<0.001
217	0.107	1.113	0.228	0.638
239	0.683	1.980	0.228	0.003
362	-1.991	0.137	0.218	<0.001
853	-1.242	0.289	0.221	<0.001
217 * 0.5% yeast	0.658	1.931	0.320	0.04
217 * 2% yeast	0.567	1.764	0.311	0.068
217 * 5% yeast	0.096	1.101	0.321	0.764
217 * 14% yeast	0.206	1.229	0.324	0.524
239 * 0.5% yeast	1.771	5.877	0.318	<0.001
239 * 2% yeast	2.119	8.326	0.305	<0.001
239 * 5% yeast	1.042	2.834	0.321	0.001
239 * 14% yeast	-0.799	0.450	0.322	0.013
362 * 0.5% yeast	3.937	51.246	0.315	<0.001
362 * 2% yeast	2.434	11.404	0.307	<0.001
362 * 5% yeast	0.691	1.995	0.318	0.03
362 * 14% yeast	-0.071	0.932	0.323	0.826
853 * 0.5% yeast	3.228	25.234	0.312	<0.001
853 * 2% yeast	2.482	11.960	0.303	<0.001
853 * 5% yeast	0.739	2.094	0.322	0.022
853 * 14% yeast	0.033	1.033	0.319	0.918

Table S8. Effect of diet on mortality within water-supplemented DGRP-195.

	Estimates from individual model			
coefficient	estimate	exp	s.e.	p
0.5% yeast	-0.512	0.599	0.098	<0.001
2% yeast	-2.124	0.120	0.100	<0.001
5% yeast	-1.322	0.267	0.102	<0.001
14% yeast	1.410	4.095	0.100	<0.001

Journal of Cell Science • Supplementary information

Table S9. Effect of diet on mortality within water-supplemented DGRP-217.

	Estimates from individual model			
coefficient	estimate	exp	s.e.	p
0.5% yeast	0.357	1.429	0.214	0.095
2% yeast	-1.487	0.226	0.215	<0.001
5% yeast	-1.011	0.364	0.216	<0.001
14% yeast	1.260	3.527	0.215	<0.001

Table S10. Effect of diet on mortality within water-supplemented DGRP-239.

	Estimates from individual model			
coefficient	estimate	exp	s.e.	p
0.5% yeast	1.114	3.046	0.119	<0.001
2% yeast	0.150	1.162	0.118	0.204
5% yeast	-0.012	0.988	0.117	0.919
14% yeast	0.096	1.101	0.118	0.413

Table S11. Effect of diet on mortality within water-supplemented DGRP-362.

	Estimates from individual model			
coefficient	estimate	exp	s.e.	p
0.5% yeast	3.631	37.764	0.386	<0.001
2% yeast	0.518	1.678	0.379	0.172
5% yeast	-0.487	0.614	0.380	0.2
14% yeast	0.920	2.509	0.379	0.015

Table S12. Effect of diet on mortality within water-supplemented DGRP-853.

	Estimates from individual model			
coefficient	estimate	exp	s.e.	p
0.5% yeast	2.811	16.623	0.222	<0.001
2% yeast	0.564	1.759	0.216	0.009
5% yeast	-0.284	0.753	0.216	0.19
14% yeast	0.923	2.518	0.216	<0.001

Journal of Cell Science • Supplementary information

Journal of Cell Science • Supplementary information

Table S13. Effect of diet and water supplementation on fecundity across 5 DGRP lines, derived from linear model estimates oflog-transformed raw fecundity counts (DGRP-195 is reference). Counts generated using QuantiFly software. Counts are relative, but directly comparable.

	Full Model			Compared to 8%	
coefficient	estimate	s.e.	p	estimate	p
intercept	2.731	0.067	<0.001		
water	0.039	0.027	0.15		
0.5% yeast	-1.374	0.092	<0.001		
2% yeast	-0.595	0.092	<0.001		
5% yeast	-0.367	0.092	<0.001		
14% yeast	0.157	0.092	0.09	2.888	<0.001
217	-0.044	0.092	0.634		
239	-0.331	0.095	0.001		
362	-0.294	0.092	0.002		
853	-0.221	0.095	0.021		
217 * 0.5% yeast	0.113	0.130	0.387		
217 * 2% yeast	-0.268	0.134	0.048		
217 * 5% yeast	-0.164	0.132	0.216		
217 * 14% yeast	-0.224	0.132	0.092	-0.110	0.404
239 * 0.5% yeast	0.509	0.139	<0.001		
239 * 2% yeast	0.310	0.139	0.027		
239 * 5% yeast	-0.119	0.134	0.373		
239 * 14% yeast	-0.262	0.136	0.056	-0.436	0.001
362 * 0.5% yeast	0.524	0.130	<0.001		
362 * 2% yeast	0.212	0.130	0.106		
362 * 5% yeast	0.180	0.130	0.168		
362 * 14% yeast	-0.202	0.130	0.122	-0.339	0.009
853 * 0.5% yeast	0.249	0.134	0.065		
853 * 2% yeast	-0.233	0.132	0.079		
853 * 5% yeast	-0.137	0.132	0.3		
853 * 14% yeast	-0.657	0.134	<0.001	-0.721	<0.001

Table S14. Effect of diet and water supplementation on fecundity across 5 DGRP lines, derived from linear model estimates oflog-transformed mortality-adjusted fecundity counts (DGRP-195 is reference). Counts generated using QuantiFly software. Counts are relative, but directly comparable.

	Full Model			Compared to 8%	
coefficient	estimate	s.e.	p	estimate	p
intercept	0.776	0.067	<0.001		
water	-0.004	0.027	0.873		
0.5% yeast	-1.379	0.093	<0.001		
2% yeast	-0.602	0.093	<0.001		
5% yeast	-0.384	0.093	<0.001		
14% yeast	0.148	0.093	0.114	0.925	<0.001
217	-0.050	0.093	0.594		
239	-0.350	0.096	<0.001		
362	-0.239	0.093	0.011		
853	-0.241	0.096	0.013		
217 * 0.5% yeast	0.101	0.132	0.444		
217 * 2% yeast	-0.301	0.136	0.028		
217 * 5% yeast	-0.113	0.134	0.398		
217 * 14% yeast	-0.165	0.134	0.218	-0.067	0.617
239 * 0.5% yeast	0.542	0.141	<0.001		
239 * 2% yeast	0.311	0.141	0.028		
239 * 5% yeast	-0.120	0.136	0.378		
239 * 14% yeast	-0.237	0.138	0.087	-0.439	0.001
362 * 0.5% yeast	0.485	0.132	<0.001		
362 * 2% yeast	0.177	0.132	0.182		
362 * 5% yeast	0.186	0.132	0.16		
362 * 14% yeast	0.166	0.132	0.209	0.075	0.568
853 * 0.5% yeast	0.284	0.136	0.038		
853 * 2% yeast	-0.227	0.134	0.091		
853 * 5% yeast	-0.111	0.134	0.406		
853 * 14% yeast	-0.550	0.136	<0.001	-0.643	<0.001

Journal of Cell Science • Supplementary information
